# Cambodia's Imminent Graduation from Least Developed Country Status: What Will be the Impact of the TRIPS Agreement on Access to HIV and Hepatitis C Medicines in Cambodia?

**DOI:** 10.1177/27551938241242602

**Published:** 2024-04-02

**Authors:** Brigitte Tenni, Joel Lexchin, Sovath Phin, Deborah Gleeson

**Affiliations:** 1 School of Public Health, 2080 La Trobe University, Melbourne, Australia; 2 Nossal Institute for Global Health, University of Melbourne, Melbourne, Australia; 3 School of Health Policy and Management, 7991 York University, Toronto, Canada; 4 Faculty of Law and Public Affairs, 231161 Pannasastra University of Cambodia (PUC), Phnom Penh, Cambodia; 5 School of Psychology and Public Health, 2080 La Trobe University, Melbourne, Australia

**Keywords:** TRIPS, intellectual property, hepatitis C, antiretroviral, direct acting antiviral, patent, generic, HIV, Cambodia

## Abstract

Cambodia has experienced exponential economic growth in recent years and is expected to graduate from least developed country (LDC) status within the next decade. Membership of the World Trade Organization (WTO) will require Cambodia to grant product and process patents for pharmaceuticals upon LDC graduation. This study aims to measure the impact of the WTO Agreement on Trade-Related Aspects of Intellectual Property Rights (TRIPS) on the price of HIV and hepatitis C medicine in Cambodia once it graduates from LDC status and is obliged to make patents available for pharmaceutical products and processes. Using scenarios based on likely outcomes of accession to the TRIPS Agreement, it measures the impact on the price of the HIV treatment program and compares that impact with the hepatitis C treatment program. Graduation from LDC status would be expected to result in a modest increase in the cost of the antiretroviral (ARV) treatment program and very large increases in the cost of the direct acting antivirals (DAA) treatment program. If annual treatment budgets remain constant, patent protection could see 1,515 fewer people living with HIV able to access ARV treatment and 2,577 fewer people able to access DAA treatment (a drop in treatment coverage of 93%).

Cambodia's rapid economic growth in the decade preceding the COVID-19 pandemic made it one of the fastest-growing economies in the region.^1^ This growth has significantly reduced the number of people living in poverty.^2^ Cambodia is currently designated a least developed country (LDC) by the United Nations with expectations of graduating from LDC status in the next decade.^3^ Cambodia's membership of the World Trade Organization (WTO) requires it to provide patent protection for pharmaceuticals that meet standard criteria upon the loss of LDC status. This obligation could potentially compromise Cambodia's access to affordable medicine^4^ and its ability to achieve its goal of universal health coverage.^5^ There is growing awareness of the need to balance Cambodia's intellectual property (IP) policies and regulation with public health priorities to ensure access to affordable lifesaving medicine, diagnostics, and vaccines to diagnose, manage, and treat a broad range of medical conditions. This research aims to measure the impact of granting patents on the price of medicine in Cambodia once it graduates from LDC status. Specifically, it measures the potential impact on the price of the antiretroviral (ARV) treatment program for HIV in comparison with the impact on the price of the hepatitis C treatment program. 

The first section of the paper outlines Cambodia's economic and political situation, its obligations with regard to membership of the WTO, and its current patent law as it applies to pharmaceuticals. It explores what Trade-Related Aspects of Intellectual Property Rights (TRIPS) flexibilities are omitted in Cambodia's IP laws and what TRIPS-plus provisions are present. It then provides an overview of the HIV and hepatitis C epidemiology and treatment programs in Cambodia before describing the methods, results, discussion, recommendations, and conclusion.

## Background

### Cambodia's Socioeconomic Situation

Cambodia's economic growth moved it into lower middle income country status^1^ in 2015. Cambodia has articulated a goal to attain upper middle-income status by 2030.^6^ These economic gains, however, are fragile, and large swaths of the economy are still living in extreme poverty. Many Cambodians live just above the poverty line, making them highly vulnerable to economic shocks.^7^ This vulnerability has been exacerbated by the COVID-19 pandemic.^8^ Increasing gross domestic product will have implications for Cambodia's eligibility for aid for health care. Many donors such as the Global Fund to Fight AIDS, Tuberculosis and Malaria and the Vaccine Alliance have already begun to reduce their funding and will continue to do so in the coming years.^9^

### Cambodia's Membership in the World Trade Organization and TRIPS

Cambodia joined the WTO in October 2004.^10^ As a member of the WTO, Cambodia is obliged to implement the 1995 WTO TRIPS Agreement, a comprehensive multilateral agreement on IP requiring baseline levels of IP protection.^11,12^ This agreement includes providing at least 20 years of patent protection for pharmaceutical products or processes that meet minimum standards of patentability, novelty, inventive step, and industrial applicability.^13^ From its inception, TRIPS was fiercely opposed by many low and middle-income countries (LMICs) who feared it would compromise their development and ability to access essential medicine.^14^ TRIPS flexibilities were included in the original text to mitigate some of the LMIC concerns. The Declaration on the Agreement TRIPS and Public Health of 2001 (The Doha Declaration) was subsequently adopted by the WTO with the aim to promote a balanced interpretation and implementation of the provisions of the TRIPS Agreement in a manner that is supportive of a WTO member's right to protect public health and promote access to medicine for all.^15^ The Doha Declaration reaffirmed the right of WTO members to implement the TRIPS flexibilities to address public health concerns and specifically recognized that patents and prices were a barrier to affordable medicine.^15^

One of these flexibilities is the transition period^2^ for LDCs. This transition period frees LDCs, such as Cambodia, from TRIPS obligations related to patents or other IP rights on pharmaceuticals until 2033 or until they are no longer an LDC^3^.^16^ Some other flexibilities available to all WTO Member States include the freedom to set patentability criteria domestically (e.g., to not make patents available for new use of known substances, methods and processes, and to use strict patentability criteria for examination of pharmaceutical patents to mitigate frivolous patents and “evergreening” opportunities); to enable pre- and post-grant opposition of patents; to use compulsory licensing and parallel importation;^4^ and to provide a Bolar (early working) exception for research and experimental use.^17,18^

Cambodia met all three criteria for LDC graduation at the most recent review in 2021^19^ and is expected to graduate from LDC status^5^ in the coming years and, subsequently, will be required to grant product and process patents for pharmaceuticals. Cambodia already has some of the highest out-of-pocket expenditure on healthcare in Southeast Asia.^20^ This change in Cambodia's status combined with the loss of external aid for health care could potentially negatively affect access to medicine.

There is a dearth of literature that investigates the impact or potential impact of LDC graduation on access to medicine in LDCs that have graduated, or where graduation is imminent. Two recent qualitative studies explored the implications of the loss of the TRIPS Agreement's LDC-specific flexibilities on the four WTO members (Cambodia, Djibouti, Senegal, and Zambia) that met the graduation criteria for the first time in 2021.^21,22^ These studies concluded that all four countries will need to reform their patent laws and policies to include all TRIPS flexibilities afforded them in order to minimize the impact of patent protection on access to medicine.^21,22^ These studies did not seek to quantify the impact of LDC graduation on medicine prices or access. They analyzed Cambodia's patent related laws and policies for only very select TRIPS flexibilities, such as compulsory licensing and the LDC TRIPS waiver, and not the full range of flexibilities afforded it.

Other studies of the impact of LDC graduation have exclusively focused on Bangladesh, given its looming 2026 graduation date and significant generic pharmaceutical industry.^23–27^ A recent study found Bangladesh's LDC graduation can be expected to significantly increase the price of insulin, which could result in a 15 percent decline in the welfare of households in Bangladesh with one or more members living with diabetes.^28^ Most of the studies have focused on the implications of graduation for Bangladesh's generic industry rather than on outcomes for access to medicine in Bangladesh. Some of these implications include high production and export costs that could undermine the sustainability of the industry.^24^

Systematic reviews of the literature that measure the impact of IP rules on access to medicine found that the introduction of patent protection leads to an increase in the price of medicine and a decrease in consumer welfare.^17,29^ Additionally, several studies from India have found that the introduction of product patent protection, following the TRIPS Agreement transition period, caused medicine prices to rise^30,31^ and resulted in large losses in consumer welfare and welfare losses for the Indian economy.^31–33^ However, there is scant research outside of India that quantifies the impact of the introduction of patent protection on access to medicine.

### Cambodia's Patent Law

Cambodia's existing patent law of 2003, The Law on Patents, Utility Model Certificates and Industrial Designs, its amendment in 2017, and its implementing regulation will govern patent grants once Cambodia graduates from LDC status. It is, therefore, critical that it includes the public health-related flexibilities and safeguards afforded by TRIPS. Cambodia's patent-related legislation omits several key public health-related TRIPS flexibilities, although a Law on Compulsory Licensing for Public Health has recently been introduced.^34^ Table 1 outlines the TRIPS flexibilities that Cambodia's patent law currently omits.

**Table 1.** TRIPS Flexibilities Omitted in Cambodia's IP Laws and Policies.

TRIPS flexibility omitted	Implication of omission
Exclusion from Patentability: Excludes new use of known substances, methods and processes (TRIPS Articles 27.2 and 27.3)	The Law on Patents, Utility Model Certificates and Industrial Designs 2003 does not specifically exclude patents for new uses of known substances, methods and processes.^35^ It therefore leaves open the opportunity for secondary patenting and granting poor quality patents. As opposed to primary patents, which protect an active ingredient, secondary patents protect a range of chemicals related to an active ingredient (such as crystalline forms of the original compound), methods of use, formulations, dosages, etc.^36^
Patentability Criteria: Develop and apply strict patentability criteria for examination of pharmaceutical patents. Mitigate frivolous patents and “evergreening” opportunities (TRIPS Articles 1 and 27.1)	The Law on Patents, Utility Model Certificates and Industrial Designs 2003 contains standard patentability criteria; however, it does not define “inventive step” in a manner that would exclude evergreening patents. Evergreening is the filing of multiple, often successive patent applications on therapeutically minor or insignificant variations of or indications for the same compound.^37^ Cambodia's lower threshold for the inventive step could result in more low-quality patents being granted.
Post-grant patent opposition: Ability to contest patents after they have been granted (TRIPS Article 62.4)	The Law on Patents, Utility Model Certificates and Industrial Designs 2003 does not provide an opportunity for post-grant opposition and any request for revocation or invalidation must be filed through a judicial court procedure.
Exceptions: Bolar (early working) exception, research and experimental use exception, individual use (TRIPS Article 30)	The Bolar exception allows for the development, testing, and experimental work necessary to obtain regulatory approval to occur while the patent is still valid. This enables generics to be ready to be manufactured and enter the market upon patent expiry of the originator medicine. Without a Bolar exception, generic firms are unable to prepare for regulatory approval until after patent expiry, which can take two or more years, and its absence extends the effective monopoly period for originator medicine.^38^
Parallel Importation (TRIPS Article 6)	Cambodia has conflicting laws on parallel importation. The Law on Patents, Utility Model Certificates and Industrial Designs 2003 allows for international exhaustion of patent rights^39^ while the Law Concerning Marks, Trade Names and Acts of Unfair Competition includes a national exhaustion doctrine, which could prohibit parallel importation.^40^

Adapted from The UNDP 2010 Good Practice Guide: Improving Access to Treatment by Utilizing Public Health Flexibilities in the WTO TRIPS Agreement.

Additionally, Cambodia's government has adopted various measures to streamline and accelerate the patent registration process that go well beyond TRIPS requirements. They include allowing the accession to multiple patent agreements designed to expedite the granting of patents,^41^ such as the Joint Statement of Intent with Japan,^42^ the Memorandum of Understanding (MoU) on Intellectual Property Cooperation with China,^43^ the MoU with Singapore,^44^ the Patent Cooperation Treaty,^45^ the MoU with South Korea,^46^ a Worksharing Arrangement with the United States,^47^ and the Patent Validation Agreement with the European Patent Organization.^48^

Cambodian patent law contains other TRIPS-plus provisions including a mailbox system.^18^ This system allows for the filing of patent product applications during the LDC transition period—despite no TRIPS requirement to do so.^35^ In accordance with Cambodia's Rule 45 of the Prakas on the Procedure for Granting Patents and Utility Model Certificates, mailbox applications will not be examined as to their patentability until 2033, or when Cambodia ceases to be a LDC.^49,50^ Cambodia's WTO Accession Agreement also contains TRIPS-plus clauses including patent linkage and data exclusivity^51^ although these provisions have not been incorporated into Cambodian legislation to date. Patent linkage refers to the conditional relationship between the granting of marketing approval of a generic medicine and the patent status of the originator medicine.^52^ Data exclusivity provides a period of time in which generic pharmaceutical companies cannot rely on existing medicine trial data submitted to regulatory authorities to obtain market approval for a generic medicine.^53^

### HIV Epidemiology and Treatment in Cambodia

In 2020, Cambodia had an HIV prevalence of 0.6 percent and approximately 75,000 people living with HIV (PLHIV).^54^ Cambodia has been extremely successful in preventing new HIV infections, which declined steadily from a high of 15,000 per year in 1995 and 1996 to 1,100 in 2020.^54^ Cambodia's National Center for HIV/AIDS Dermatology and STDs rapidly scaled up the provision of ARV treatment from 2001.^55^ By the end of 2020, a total of 63,338 PLHIV were accessing ARV treatment, representing 84 percent treatment coverage of PLHIV.^54^ See Table 2 for the number of PLHIV on first-, second-, and third-line regimes and the ARVs included in these regimes. In 2020, 77 percent of ARV costs were covered by the Global Fund to fight AIDS, Tuberculosis and Malaria (GFATM) and 23 percent from Cambodian government sources.^56^ Domestic funding for ARV treatment has steadily increased from $708,000 in 2015 to $1.25 million in 2020 (all dollar amounts in U.S. dollars), however Cambodia is still heavily reliant on external donor funding for its ARV treatment program.^56^

**Table 2.** ARV Treatment Regimen and Number of PLHIV on Each Regimen in Cambodia.

Pharmaceutical Data	Number of people accessing treatment (2020)
Number of adults living with HIV on each ARV regimen	
1st line regimen	
Tenofovir/Lamivudine/Efaviranz - 300/300/400mg - TDF/3TC/EFV	31,923
Tenofovir/Lamivudine/Dolutegravir - 300/300/50mg - TDF/3TC/DTG	20,939
Other regimen	4,888
2nd line regimen	
Tenofovir/Lamivudine (300/300mg) + Atazavir/ritonavir (300/100mg) - (TDF/3TC + ATV/r)	2,748
Tenofovir/Lamivudine (300/300mg) + Lopinavir/ritonavir (200/50mg) - (TDF/3TC + LPV/r)	120
Zidovudine/Lamivudine (300/150mg) + Atazavir/ritonavir (300/100mg) - AZT/3TC + ATV/r	454
Zidovudine/Lamivudine (300/150mg) + Lopinavir/ritonavir (200/50mg) - AZT/3TC + LPV/r	64
Abacavir/Lamivudine (600/300mg) + Atazanavir/ritonavir (300/100mg) - ABC/3TC + ATV/r	527
Abacavir/Lamivudine (600/300mg) + Lopinavir/ritonavir (200/50mg) - ABC/3TC + LPV/r	78
Other regimen	44
3rd line regimen	
Darunavir 600mg + TDF/3TC/DTG + Ritonavir 100mg	32
Darunavir 600mg + Dolutegravir 50mg + Lamivudine 150mg + Ritonavir 100mg	14
Darunavir 600mg + Dolutegravir 50mg + Ritonavir 100mg	9
Other regimen	22
Number of adults living with HIV on ARV treatment in total	61,862

Compiled using National Center for HIV/AIDS, Dermatology and STD 2020 data.

### Hepatitis C Epidemiology and Treatment in Cambodia

Hepatitis C is a blood-borne virus that, untreated, can lead to liver damage and diseases, such as cirrhosis and liver cancer, and death.^57^ Although there is no official data on the number of people living with hepatitis C in Cambodia, Médecins Sans Frontières (MSF) estimates that 2–5 percent of the population is infected.^58^ MSF, together with the Cambodian Ministry of Health, launched Cambodia's first free hepatitis C treatment program in May 2016 at Preah Kossamak hospital in Phnom Penh in line with the World Health Organization's (WHO) global goal to eliminate hepatitis C by 2030.^59^ Treatment was fully funded by MSF and consisted of a 12-week course of direct acting antivirals (DAA), which has a cure rate of 97 percent.^59^

In 2020, 2,776 people completed hepatitis C treatment provided by MSF/Ministry of Health. This treatment program relies heavily on generic medicines made possible by Cambodia realizing its right as a LDC to not grant patents for pharmaceuticals. The treatment regime consisted of two generic DAAs in combination, 400mg of sofosbuvir and 60mg of daclatasvir, given once a day for 12 weeks. They were sourced from the generic companies Mylan, Natco, Pharco, and Hetero. This pan-genotypic regime was chosen because most hepatitis C infections in Cambodia are genotype 1 and 6 and a small number are genotype 2, which all respond well to the sofosbuvir/daclatasvir regime and do not require genotype testing. The cure rate was also very high for patients with liver cirrhosis, with an estimated 90 percent cure rate for patients with compensated cirrhosis and 66 percent for patients with decompensated cirrhosis.^60^

### Aim and Research Questions

This study aims to measure the potential impact of the TRIPS Agreement on the price of medicines in Cambodia once it graduates from LDC status and is obligated to make patents available for pharmaceutical products and processes. Specifically, it measures the anticipated impact on the ARV treatment program where most ARVs are off-patent and compares that with the impact on the hepatitis C treatment program where many medicines are still under patent in high-income countries. Impact is measured in terms of the cost of each treatment program and the number of people able to access treatment. The research questions addressed are:

- What is the potential impact of Cambodia's implementation of TRIPS following LDC graduation on access to ARV treatment for HIV, measured by the cost of the ARV program and the number of people able to access treatment?
- What is the potential impact of Cambodia's implementation of TRIPS following LDC graduation on access to DAA for hepatitis C, measured by the cost of the hepatitis C treatment program and the number of people able to access treatment?
- How do the impacts on these treatment programs compare, and how can any differences be explained?

## Methods

### Scenario Development

Three possible ARV scenarios and two possible DAA scenarios were designed to explore the range of potential impacts of the TRIPS Agreement on Cambodia's ARV and DAA treatment programs. Only two DAA scenarios were explored as it was assumed that there would be no difference between baseline and full TRIPS flexibilities as Scenario 1 used only generic medicine (see Table 3 for the scenarios and their assumptions).

**Table 3.** ARV and DAA Scenarios and Their Assumptions.

	Scenario 1 (baseline) - Current situation given LDC status and LDC TRIPS waiver	Scenario 2- Introduction of patents when Cambodia graduates from LDC status and the LDC TRIPS waiver no longer applies	Scenario 3 - Introduction of patents when Cambodia graduates from LDC status, with full implementation of TRIPS flexibilities in its domestic law
ARV Scenarios			
Assumptions	ARV regimens outlined in Table 2 used in Cambodia in 2020 NCHADS prices for these regimens	ARV regimens outlined in Table 2 used in Cambodia in 2020 LDC status no longer applies and Cambodia is required to grant patents in accordance with TRIPS requirements Cambodia's mailbox contains patent applications for ARVs that had previously been granted patents in the European Union, Japan, Singapore, United States, China, and/or South Korea	ARV regimens outlined in Table 2 used in Cambodia in 2020 Cambodia's patent law allows for compulsory licensing of all patented medicine in the national ARV treatment program Cambodia is then able to import generic versions of these patented ARVs at the lowest global prices
DAA Scenarios			
Assumptions	DAA regimen consisting of the sofosbuvir and daclatasvir regimen used by MSF-France in Cambodia in 2020	LDC status no longer applies and Cambodia is required to grant patents in accordance with TRIPS requirements Cambodia's mailbox contains patent applications for DAAs that had previously been granted patents in the European Union, China, Japan, Singapore, United States, and/or South Korea

NCHADS: National Center for HIV/AIDS, Dermatology and STD.

### Data Collection

The number of people treated with ARVs in Cambodia in 2020 was sourced from National Center for HIV/AIDS, Dermatology and STD (NCHADS) and was a constant for all three scenarios. The price data for ARV Scenario 1 was also sourced from NCHADS. Price data for the second and third ARV scenarios was sourced from the MSF publication, “Untangling the Web: HIV Medicine Pricing & Access Issues, 2020”.^61^ This periodic publication provides an update on the availability, price, and supplier information in relation to ARVs globally. It details the lowest generic ARV prices available globally, which was relevant for Scenario 3 and details what the originator brands would charge Cambodia for lopinavir/ritonavir, darunavir and dolutegravir, the three ARVs that would be patented under the assumptions outlined in Scenario 2.

The number of people treated with DAAs in Cambodia in 2020 was provided by MSF and was constant for Scenario 1 and 2. The price data for DAA Scenario 1 was also sourced from MSF. The DAA price data for Scenario 2 was sourced from the WHO publication “Accelerating Access to Hepatitis C Diagnostics and Treatment: Overcoming Barriers in Low and Middle-Income Countries – Global Progress Report 2020”.^62^ This publication provides updated data on pricing, licensing, and regulatory status of hepatitis C virus (HCV) medicines from the main originator and generic companies producing DAAs for HCV treatment. The lowest reported originator price for daclatasvir was sourced from this publication and used in DAA Scenario 2. The reported low-income country price for sofosbuvir was used for Scenario 2 and was also sourced from this publication.

The ARV and DAA data were entered into an Excel spreadsheet (see Supplementary File 1).

The World Intellectual Property Organization's Patent Scope database and the European Patent Office website were scanned for possible patents on ARVs included in the first-, second-, and third-line regimes and for DAAs used in the 2020 Cambodian treatment program (see Tables 4 and 5). It was assumed that a patent would also be granted in Cambodia if there was an existing patent in Japan, the United States, the European Union, China, Singapore, or South Korea on any of the ARVs and DAAs listed in the Cambodian treatment program and that the application for this patent was in the mailbox system. Although existing patent agreements with the above countries may vary in detail, they all obligate Cambodia to accelerate or grant pharmaceutical product patents if they had been granted in one or more of these countries once Cambodia is required to grant patents and those existing patent agreements are still valid and implemented.

**Table 4.** ARVs Under Patent in Countries with Which Cambodia has a Patent Agreement.

ARV	Class of ARV	Patent holder	Originator brand name
Lopinavir/ritonavir	Protease inhibitor	AbbVie	Kaletra, Aluvia
Darunavir	Protease inhibitor	Janssen-Cilag	Prezista
Dolutegravir	Integrase inhibitor	ViiV Healthcare	Tivicay

Compiled from Patentscope database and European Patent Office website.

**Table 5.** DAA Under Patent in Countries with Which Cambodia has a Patent Agreement.

DAA	Patent holder	Originator brand name
Daclatasvir	Bristol Myers Squibb	Daklinza
Sofosbuvir	Gilead Sciences, Inc.	Sovaldi

Compiled from Patentscope database and European Patent Office website.

### Data Analysis

The cost of the ARVs and DAAs was calculated for each of the scenarios by multiplying the number of adults requiring a particular treatment regime by the unit cost of the medicine and adjusting for dosing (see Supplementary File 1 for full calculations). Using ARV Scenario 1 as a fixed total budget, the percentage of people who could access ARV treatment given the cost of each scenario was calculated. These percentages were compared for the ARV scenarios. The number of people able to be treated in each of the ARV scenarios using the budget for Scenario 1 was also calculated and compared. The cost of the DAA scenarios were calculated and compared. The percent change in the number of people able to access DAAs in Scenario 2 was calculated.

## Results

### Patent Status of ARVs and DAAs

ARVs found to be still under patent in any of the countries with which Cambodia has a patent agreement (European Union, China, Japan, Singapore, United States, and South Korea) are detailed in Table 4. The ARVs under patent (detailed in Table 4) form part of the second- or third-line regimens.

Both DAAs used in the treatment program in 2020 were found to be under patent in one or more of these jurisdictions. They are detailed in Table 5.

### Costs Associated with Each Scenario

Table 6 details the cost of the three ARV scenarios. Detailed workings are shown in Supplementary File 1.

**Table 6.** Costed ARV Scenarios.

ARV scenarios			
	Scenario 1 (baseline) - Current situation given LDC status and LDC TRIPS waiver	Scenario 2- Introduction of patents when Cambodia graduates from LDC status and the LDC TRIPS waiver no longer applies	Scenario 3-Introduction of patents when Cambodia graduates from LDC status, with full implementation of TRIPS flexibilities in its domestic law
Total cost of each scenario	$4,915,960	$5,039,461	$4,685,456
Percentage of PLHIV covered by cost of scenario	84%	82%	88%
Number of people covered by cost of Scenario and fixed baseline budget	61,826	60,311	64,906

### ARV Scenarios

The most expensive ARV scenario is Scenario 2 ($5,039,461), which is marginally more expensive than Scenario 1 (baseline; $4,915,960). The least expensive scenario is Scenario 3 ($4,685,456), where the Cambodian government purchases the cheapest generics on the market. Treatment coverage would drop by 2 percent to 82 percent in Scenario 2 and increase by 4 percent to 88 percent in Scenario 3 if the existing Scenario 1 budget remained unchanged. Given the existing budget detailed in Scenario 1, Scenario 2 would see 1,515 fewer PLHIV able to access ARV. Scenario 3 would allow an additional 3,080 PLHIV to access ARVs given the baseline budget.

### DAA Scenarios

The most expensive DAA scenario is Scenario 2 ($3,431,136). It is 14 times more expensive than Scenario 1 (baseline; $245,676). Given the budget of Scenario 1 and the cost of Scenario 2, the latter would see a 93 percent reduction in the number of people with hepatitis C able to access DAA treatment. Given the existing budget detailed in Scenario 1, Scenario 2 would see 2,577 fewer people able to access DAAs. Table 7 details the cost of the DAA scenarios.

**Table 7.** Costed DAA Scenarios.

DAA regime			
	SOF	DCV	Total
Scenario 1 (baseline) - Current situation given LDC status and LDC TRIPS waiver			
Cost of DAA regime per person	$52.50	$36	
Number of people treated	2,776	2,776	
Total cost of DAA regime by number of people treated	$145,740	$99,936	$245,676
Scenario 2- Introduction of patents when Cambodia graduates from LDC status and the LDC TRIPS waiver no longer applies			
Cost of DAA regime per person	$690	$546	
Number of people treated	2,776	2,776	
Total cost of DAA regime by number of people treated	$1,915,440	$1,515,696	$3,431,136

Abbreviations: SOF, sofosbuvir; DVC, daclatasvir.

## Discussion

### Impact of TRIPS

These results demonstrate that Cambodia's future TRIPS obligations to grant patents could potentially give rise to modest increases in the cost of the ARV program and significant increases in the cost of the DAA treatment program. Anticipated costs would be only marginally higher for the ARV treatment program under a patent protection regime as most ARVs in current use are older medicines and largely off-patent in most jurisdictions. Newer patented ARVs tend to only be used in second- and third-line regimens that have far fewer enrolments. These regimens are used only when a PLHIV has become resistant to a first-line regime or there are other clinical indications to avoid medication in a first-line regimen. However, more and more PLHIV are likely to transition to these regimens in the future. Additionally, it is possible that the current first-line regimen will be superseded by newer, more effective, or safer ARVs in the future. New ARVs are almost certain to be patented and, therefore, cost more than current off-patent first-line generics, thereby increasing the cost of the treatment regimen and possibly leading to fewer people being able to access treatment. These findings are consistent with studies that have explored the impact of the introduction of patents following the end of the transition period on medicine prices in India.^30–33^

### Increased Cost to the ARV and DAA Treatment Program

Despite the small differences in coverage between the scenarios, the increased costs due to patent protection could result in 1,515 fewer PLHIV able to access treatment given the current budget. This reduction in coverage is not inconsiderable given that Cambodia's graduation from LDC status could also mean a reduction in external funding for the HIV program and ARVs, specifically. Eligibility for funding from the GFATM is determined by a country's income classification and disease burden. Cambodia is currently classified as having a high HIV disease burden and has recently been classified a LMIC by the World Bank. Cambodia will lose eligibility for GFATM funding, its only external funder for ARVs, if its HIV burden is not deemed high and if it transitions to upper-middle income country status.

Although ARVs are not ideal medicines to use to measure the impact of patent protection, they provide a useful comparison to newer and, therefore, more likely to be patented medicines, such as DAAs. The DAA scenarios did show considerable difference in cost given the high prices charged by the patent holders, Gilead and Bristol Myers Squibb (BMS). In fact, DAAs have proven to be incredibly lucrative for Gilead and BMS. When Gilead first launched sofosbuvir as Sovaldi in 2014, a 12-week course of treatment cost $84,000 to $94,000, or approximately $1,000 per pill.^63^ In its first year on the market, Sovaldi generated $10.3 billion in sales for Gilead and came close to being the highest revenue-producing medicine in the world.^64^ Cambodia may be forced to pay these inflated prices if it is unable to access generic alternatives legally. Generic competition has reduced the price of DAAs dramatically in jurisdictions where they are not under patent.

MSF's presence in Cambodia ended in December 2021 when they handed over financial and managerial responsibility of the hepatitis C treatment program to the Cambodian government. Unfortunately, the Ministry of Health is yet to secure a budget for DAA procurement; currently only those able to pay for treatment in the private sector can access DAAs. Treatment with generic DAAs is estimated to cost approximately $600 for a 12-week course.^65^ The Ministry of Health hopes to be able to secure funding for DAA treatment in 2023. This is a critical time for Cambodia as looming patent protection may compel Cambodia to pay considerably more for DAAs at a time when it assumes full financial responsibility from MSF for the hepatitis C treatment program.

### The Limits of Voluntary Licensing as a Remedy for Patent Barriers

It has been argued that IP barriers, such as the introduction of patent protection, can be addressed by remedial policies like voluntary licensing.^66^ A voluntary license (VL) is an authorization given by a patent holder to a generic company, allowing it to produce a generic version of a patented pharmaceutical product.^67^ VLs have been touted as a way to address the barriers that patents pose such as high prices and can substantially improve access to affordable generic medicines^68^; however, current VL agreements would not provide sufficient access to the suite of medicines needed for Cambodia's HIV and hepatitis C treatment programs to alleviate the effects of Scenario 2 for each program.

It is possible that VL could enable Cambodia to access generic dolutegravir for HIV even if the company marketing it is granted patents under the conditions described in Scenario 2. The patent holder of dolutegravir, ViiV Healthcare, has signed VL agreements with the Medicines Patent Pool (MPP) that sublicenses generic manufacturers to allow them to produce and distribute generic versions of these products under the terms of the license. The MPP is a United Nations-backed public health organization working to increase access to and facilitate the development of life-saving medicine for LMICs via VL agreements.^69^

There is also an MPP VL with the U.S. National Institutes of Health for patents related to the HIV medicine darunavir (DRV). This license does not include all patents related to DRV and it is therefore likely that Cambodia will still face IP barriers to accessing generic DRV.^70^ The original AbbVie 2014 MPP license agreement for lopinavir/ritonavir included Cambodia but was limited to pediatric formulations.^71^ The 2015 MPP AbbVie Agreement for the adult formulation of lopinavir/ritonavir was limited to African countries.^72^ In March 2020, at the onset of the COVID-19 pandemic, the Israeli Ministry of Health issued a compulsory license for lopinavir/ritonavir as a possible treatment for COVID-19. The following day, AbbVie announced that it would cease to enforce its patents on lopinavir/ritonavir anywhere in the world highlighting the importance of TRIPS flexibilities in facilitating equitable access to medicine and ensuring Cambodia will be able to access generic lopinavir/ritonavir.^73^

The VL picture is also patchy for hepatitis C medicine. BMS, the company that holds the patent for daclatasvir, also signed a VL agreement with the MPP in 2015, which enabled the generic manufacture and sale of daclatasvir in 112 LMIC. To date, one Bangladeshi and six Indian generic companies have signed sublicense agreements with BMS to manufacture and supply generic daclatasvir.^74^ Although the VL agreement fortunately includes Cambodia, it covers only 65 percent of people living with HCV in LMICs and may exclude Cambodia once it graduates from LDC status.

In addition, Gilead, which holds the patent for sofosbuvir, has signed bilateral VL agreements with 11 Indian generic manufacturers and one each in Pakistan and Egypt that allow for the manufacture and sale of generic sofosbuvir in over 100 countries,^75^ including Cambodia. It is not known what price these companies would charge Cambodia for sofosbuvir; however, it is unlikely to match the lowest price available globally. Graduation to middle-income status may change the eligibility of Cambodia to be included in some of the more restrictive VLs. Some VLs may only include LICs, and graduation from LDC status may exclude Cambodia from some of the more exclusive licenses at a time when it must implement patent protection for pharmaceuticals. For example, the ViiV dolutegravir license has been extended twice to expand the territories included, but the original 2014 agreement covered all LDC, low-income countries, and African countries royalty-free and only six LMICs were subject to royalty payments.^76^

VLs have been criticized for their lack of transparency, restrictive conditions and higher prices than what can be achieved via compulsory licensing.^67^ Access to the generics will depend on whether included generic manufacturers choose to register their products in Cambodia and what prices they command. VLs that lock in supply from specific overseas-based generic companies undermine opportunities for building domestic pharmaceutical manufacturing capacity.

Cambodia has some generic pharmaceutical companies with the potential for expansion. The COVID-19 pandemic has highlighted the need for countries to invest in their own pharmaceutical manufacturing capacity to ensure an affordable and sustainable supply of health products, including medicine. In response to supply and distribution concerns, Cambodian Prime Minister Hun Sen recently granted permission to a local generic company to produce molnupiravir (Lagevrio), an antiviral medicine used in the treatment of COVID-19.^77^ In addition, the Cambodian generic pharmaceutical company, Cambodia Pharmaceutical Enterprise, has registered and is manufacturing generic versions of daclatasvir and sofosbuvir in Cambodia.^78^ Despite these initiatives the authors could find no overarching strategy for domestic pharmaceutical manufacturing in Cambodia.

### Cambodia's Need to Reform IP Laws

To ensure it can continue to protect access to medicine, Cambodia will need to make use of the TRIPS waiver transition period to update its IP-related laws to ensure that when it is compelled to grant patents, it has incorporated all flexibilities afforded it by the TRIPS Agreement. This will minimize the impact that patent protection will have on the cost of medicine and can facilitate access to affordable generics as evidenced by the relative affordability of ARVs in Scenario 3 (in comparison to the other two ARV scenarios) that used compulsory licensing to procure the cheapest generic ARVs on the market. The current Cambodian IP governance system lacks key provisions that could result in a greater number of poor-quality patents, less opportunity to oppose them, greater cost, and longer patent monopoly periods.^18^

Cambodia's patent law unnecessarily provides for secondary patents, which add years to patent terms and delays the entry of more affordable generic medicine.^79,80^ Secondary patenting has led to increased medicine costs^81^ and large social welfare losses.^82^ The absence of a Bolar provision (which is omitted from Cambodian patent law) can add two or more years to regulatory approval times, which extends the effective monopoly period for originator medicine.^38^ Studies have shown that without parallel importation, another important TRIPS flexibility, there would be an increase in governmental health care expenditure and a decrease in consumer welfare.^83^ Parallel importation has also been shown to reduce prices for patented medicines.^83–85^ Although Cambodia has international exhaustion of patent rights that allows for parallel importation in their Law on Patents, Utility Model Certificates and Industrial Designs 2003, Cambodia's Law Concerning Marks, Trade Names and Acts of Unfair Competition includes a national exhaustion doctrine that could prevent parallel importation.^29^

A lack of an administrative post-grant opposition procedure—also reflected in Cambodian law—can deter challenges to low-quality patents due to the necessary costs and legal representation required. This absence can lengthen the cost and time taken to revoke a patent granted in error and increase patent monopoly periods. Post-grant administrative procedures can decrease the number of low-quality patent applications.^86^ Operating a mailbox system for the filing of patents without a requirement to do so will lead to patents being granted (from the filing date), which would otherwise not have been granted if pharmaceutical companies had to wait until the end of the TRIPS waiver period to submit an application. As a developing, but not least developed country, India was granted a ten-year transition period in which to implement the TRIPS Agreement and introduce patent protection for pharmaceutical products. Unlike Cambodia, India was legally required to operate a mailbox facility during this transition period. The Indian government designed Section 11A(7) of the Indian Patent Act to minimize the impact of a mailbox system. This legislation allows for the continued production of a patented product that was already in use or if there was significant investment in the production and marketing of a product prior to the introduction of patent protection, subject to “a reasonable royalty”.^87^^, p.17^ If Cambodia were to maintain the mailbox system, it could consider adopting an approach similar to India's to ensure it could maximize access to generic medicine already in use.

### Limitations

Prospective studies have been shown to find larger negative effects of trade-related IP provisions on medicine prices and costs than retrospective studies.^29^ As this is a prospective analysis, it is possible that assumptions made in this study overestimate the impact of the implementation of TRIPS obligations to grant patents for pharmaceuticals. Although it is likely that the obligation to grant patents for pharmaceuticals will impact the price of other patented medicines, the findings are valid for DAAs and ARVs only and not necessarily generalizable to other drugs. The effects of patent protection could be much greater for more expensive biologic drugs such as monoclonal antibodies and some vaccines. This is a limited analysis of the immediate effects of patent protection and does not measure the full impact of Cambodia's omission of key TRIPS Agreement flexibilities, such as parallel importation, Bolar provision, and post-grant opposition. These omissions could have more cumulative effects over time and their impact on the price of and access to medicine may not be immediately apparent. Further research is needed to better understand the impact of these flexibilities on access to medicine.

## Recommendations and Conclusions

When the LDC waiver no longer applies to Cambodia, this country will be required to implement patent provision for pharmaceuticals in its patent law. The obligation to grant patents for pharmaceuticals can be expected to lead to modest increases in the cost of the ARV treatment program due to most ARVs being off-patent worldwide and very large increases in the cost of the DAA treatment program where many medicines are still under patent. Without an increase in annual treatment budgets, patent protection could see a 2 percent drop in treatment coverage, or 1,515 fewer PLHIV able to access treatment, and 2,577 or 93 percent fewer people able to access DAA treatment. These findings suggest that graduation from LDC status and the subsequent TRIPS obligation to provide patent protection can challenge a country's ability to access affordable medicine to maintain national treatment programs.

To minimize the impact of these potential cost increases and to achieve the Ministry of Health and WHO goal of hepatitis C elimination, it is recommended that Cambodia make careful use of its transition period to ensure its patent law is inclusive of all TRIPS flexibilities and reconsider membership in patent agreements and MoUs designed to expedite the granting of patents. It is recommended that Cambodia remove the mailbox facility to avoid granting retrospective patents and being overwhelmed by patent applications. If it decides to keep the mailbox facility, it should consider including a clause similar to the one in the Indian Patent Act that permits the continued production of a patented product where it is already in use or if there was significant investment in its production and marketing prior to the introduction of patent protection. Additionally, Cambodia should amend its Law Concerning Marks, Trade Names and Acts of Unfair Competition to allow for parallel importation and the Law on Patents, Utility Model Certificates and Industrial Designs 2003 to include provisions for Bolar and post-grant opposition and tighten its patentability criteria to exclude secondary and poor-quality patents. Although unlikely, Cambodia should consider delaying graduation from LDC status until 2033 to maximize the time for patent-free access to medicine.

Further research is needed to determine the potential impact of LDC graduation on access to medicine beyond ARVs and DAAs. Qualitative research to explore how countries can best navigate the complexities of the TRIPS/access to medicine nexus and preserve access to medicine in the context of economic pressures is also vitally important.

## Supplemental Material

sj-docx-1-joh-10.1177_27551938241242602 - Supplemental material for Cambodia's Imminent Graduation from Least Developed Country Status: What Will be the Impact of the TRIPS Agreement on Access to HIV and Hepatitis C Medicines in Cambodia? Supplemental material, sj-docx-1-joh-10.1177_27551938241242602 for Cambodia's Imminent Graduation from Least Developed Country Status: What Will be the Impact of the TRIPS Agreement on Access to HIV and Hepatitis C Medicines in Cambodia? by Brigitte Tenni, Joel Lexchin, Sovath Phin and Deborah Gleeson in International Journal of Social Determinants of Health and Health Services

## List of Abbreviations

ABC: Abacavir
ARV: antiretroviral (medicine)
ATV: Atazavir
AZT: Zidovudine
DAA: direct acting antiviral (medicine)
DRV: Darunavir
DTG: Dolutegravir
EFV: Efavirenz
IP: intellectual property
LDC: least developed country
LMIC: low- and middle-income country
LPV/r: Lopinavir/ritonavir
MoU: memorandum of understanding
MPP: Medicines Patent Pool
MSF: Médecins Sans Frontières
NCHADS: National Center for HIV/AIDS, Dermatology and STD
PLHIV: people living with HIV
TDF: Tenofovir
TRIPS: Trade-Related Aspects of Intellectual Property Rights
VL: voluntary license
3TC: Lamivudine.

## Notes

1. This is a World Bank classification that assigns the world's economies to four income groups—low, lower-middle, upper-middle, and high-income countries. The classifications are updated each year on July 1 and are based on gross national income per capita in current USD of the previous year. This is different to the country economic classification system used by the WTO which uses the United Nations country economic classification system and classifies Cambodia as a least developed country (LDC).

2. In view of the special needs and requirements of least-developed country Members, their economic, financial and administrative constraints, and their need for flexibility to create a viable technological base, such Members shall not be required to apply the provisions of this Agreement, other than Articles 3, 4 and 5, for a period of 10 years from the date of application as defined under paragraph 1 of Article 65. The Council for TRIPS shall, upon duly motivated request by a least-developed country Member, accord extensions of this period.^12^

3. This transition period is the only LDC-specific TRIPS flexibility that is relevant to pharmaceuticals.

4. Parallel importation is the importation of a patented medicine (presumably at a cheaper price) without the explicit permission of the patent owner.

5. LDC status is based on three criteria: (a) per capita gross national income, (b) a human assets index, and (c) an economic vulnerability index. These criteria are reviewed every three years, most recently in 2021. A country graduates when it meets the threshold in two of the three criteria. Countries may also graduate based on gross national income alone if per capita gross national income is more than double the graduation threshold.

## References

[1] Asian Development Bank. Cambodia and ADB 2023 [cited 2023 6/10/2023]. Available from: https://www.adb.org/countries/cambodia/overview#:∼:text=ADB's%20Work%20in%20Cambodia&text=Cambodia's%20economic%20growth%20averaged%20over,growing%20economies%20in%20the%20region.

[2] World Bank. Cambodia: Reducing Poverty and Sharing Prosperity October 29, 2019 [cited 2022 9th August 2022]. Available from: https://www.worldbank.org/en/results/2019/10/30/cambodia-reducing-poverty-and-sharing-prosperity.

[3] de Gaudemar M . Cambodia to remain among Least Developed Countries, for now. The Phnom Penh Post. 09 December 2016.

[4] Doha WTO Ministerial 2001: TRIPS WT/MIN(01)/DEC/2 20 November 2001, (Adopted on 14 November 2001).

[5] Kingdom of Camboida. HEALTH STRATEGIC PLAN 2016-2020 “Quality, Effective and Equitable Health Services”. In: Department of Planning & Health Information, editor. May 2016.

[6] The World Bank. The World Bank in Cambodia- Overview 2022 [cited 2022 10th August 2022]. Available from: https://www.worldbank.org/en/country/cambodia/overview.

[7] OECD Development Pathways. Social protection system review of Cambodia. Paris: OECD Publishing; 2017.

[8] United Nations Department of Economic and Social Affairs. Lifeline for vulnerable Cambodians as poverty rises during COVID-19 pandemic [cited 2022 16/11/2022]. Available from: https://sdgs.un.org/partnerships/lifeline-vulnerable-cambodians-poverty-rises-during-covid-19-pandemic.

[9] Pearson M Mundy J . Graduating from global health programme financing: Lessons and future challenges in the asia pacific region. Mott MacDonald. 2015.

[10] World Trade Organization. Cambodia raises WTO membership to 148 2021 [cited 2022 10th August 2022]. Available from: https://www.wto.org/english/news_e/news04_e/cambodia_148members_13oct04_e.htm.

[11] World Trade Organization. Frequently asked questions about TRIPS [ trade-related aspects of intellectual property rights ] in the WTO 2021 [cited 2021 23rd February 2021]. Available from: https://www.wto.org/english/tratop_e/trips_e/tripfq_e.htm.

[12] TRIPS: Agreement on Trade-Related Aspects of Intellectual Property Rights., Annex 1C, 1869 U.N.T.S. 299, 33 I.L.M. 1197 (Apr. 15, 1994).

[13] World Trade Organization. TRIPS: DRUG PATENTS, TECHNICAL NOTE. Pharmaceutical patents and the TRIPS Agreement 2021 [cited 2021 18th March 2021]. Available from: https://www.wto.org/english/tratop_e/trips_e/pharma_ato186_e.htm?platform=hootsuite.

[14] Pacón AM . What will TRIPS do for developing countries. In: From GATT to TRIPS–the agreement on trade-related aspects of intellectual property rights, studies in industrial property and copyright law. VCH Verlagsgesellschaft mbH;1996:329–356.

[15] The World Health Organization. The Doha Decalration on the TRIPS Agreement and Public Health 2021 [cited 2023 13/9/203]. Available from: https://www.who.int/medicines/areas/policy/doha_declaration/en/.

[16] United Nations. LDC Portal International Support Measures for Least Developed Countries- WTO drugs patent waiver for LDCs extended until 2033 [Available from: https://www.un.org/ldcportal/wto-drugs-patent-waiver-for-ldcs-extended-until-2033/.

[17] Tenni B Moir HV Townsend B , et al. What is the impact of intellectual property rules on access to medicines? A systematic review. Global Health. 2022;18(1):1–40. 35428250 10.1186/s12992-022-00826-4 PMC9013034

[18] Tenni B Lexchin J Phin S Kittitrakul C Gleeson D . Lessons from India and Thailand for Cambodia's future implementation of the TRIPS Agreement for pharmaceutical patents. J World Intellect Prop. 2023;26:166–194. 10.1111/jwip.12267

[19] United Nations, Department of Econmic and Social Affairs, Committee for Development Policy. The Least Developed Country Category: 2021 Country Snapshots. 2021.

[20] Ir P Jacobs B Asante AD , et al. Exploring the determinants of distress health financing in Cambodia. Health Policy Plan. 2019;34(Supplement_1):i26–i37. 10.1093/heapol/czz006 PMC6807511 31644799

[21] Syam N Syed S . Impacts of LDC graduation on trade-related aspects of intellectual property rights (TRIPS) in Cambodia, Djibouti, Senegal and Zambia. United Nations, Department of Economics and Social Affairs; 2023.

[22] UN Committee for Development Policy. Potential impacts of LDC graduation Cambodia, Comoros, Djibouti, Senegal and Zambia. February 2023.

[23] Rahman M . Graduating from the LDC group: Challenges facing Bangladesh. South Centre; 2023.

[24] Rahman M Farin SM . Research report 2: WTO decision on TRIPS and public health: A window of opportunity for Bangladesh’s pharmaceutical industry. Center for Policy Dialogue; 2018. Also available at https://cpd. org. bd/wp …

[25] Gay D . Pharmaceutical dreams: TRIPS and drugs policy in Bangladesh. United Nations Committee for Development Policy New York. 2018.

[26] Gay D Gallagher K . The need to extend the WTO TRIPS pharmaceuticals transition period for LDCs in the COVID-19 era: Evidence from Bangladesh. CDP Policy Review No 10 United Nations Department of Economic and Social Affairs: Economic Analysis. 2020.

[27] Razzaque MA Rabi RI Akib H . Bangladesh’s Pharmaceutical Exports: Trends, Market Prospects, and Policies’. Navigating New Waters: Unleashing Bangladesh’s Export Potential for Smooth LDC Graduation. 377.

[28] Islam MD Kaplan WA Wirtz VJ Gallagher KP . The social costs of success: The impact of world trade organization rules on insulin prices in Bangladesh upon graduation from least developed country status. Asian Dev Rev. 2022;39(01):239–279.

[29] Islam MD Kaplan WA Trachtenberg D Thrasher R Gallagher KP Wirtz VJ . Impacts of intellectual property provisions in trade treaties on access to medicine in low and middle income countries: A systematic review. Global Health. 2019;15(1):88. 31888688 10.1186/s12992-019-0528-0 PMC6937733

[30] Duggan M Garthwaite C Goyal A . The market impacts of pharmaceutical product patents in developing countries: Evidence from India. Am Econ Rev. 2014;106(1):99–135.

[31] Watal J . Pharmaceutical patents, prices and welfare losses: Policy options for India under the WTO TRIPS agreement. World Econ. 2000;23(5):733–752.

[32] Chaudhuri S Goldberg PK Jia P . Estimating the effects of global patent protection in pharmaceuticals: A case study of quinolones in India. Am Econ Rev. 2006;96(5):1477–1514. 29135209 10.1257/aer.96.5.1477

[33] Dutta A . From free entry to patent protection: Welfare implications for the Indian pharmaceutical industry. Rev Econ Stat. 2011;93(1):160–178.

[34] Law On Compulsory Licensing for Public Health (2018).

[35] Rule 45 of the Prakas (declaration) No 766 MINE.DIP.PRK on the procedure for granting patents and utility model certificates.

[36] World Intellectual Property Organization. Committee on Development and Intellectual Property (CDIP) Study on Pharmaceutical Patents in Chile Geneva, Switzerland; April 20 to 24, 2015.

[37] World Health Organization. Report of the United Nations Secretary General's High Level Panel on Access to Medicines. September 2016.

[38] Munoz Tellez V . Bolar exception. In: Access to medicines and vaccines. Springer; 2022:135–149.

[39] Law on Patents, Utility Model Certificates and Industrial Designs (22 January 2003).

[40] Law Concerning Marks, Trade Names and Acts of Unfair Competition (2002).

[41] Phin S . Procedure for Patent Registration in Cambodia: In-House Community; August 2, 2017 [cited 2021 26th March 2021]. Available from: https://www.inhousecommunity.com/article/procedure-patent-registration-cambodia.

[42] Joint Statement of Intent on Cooperation for Facilitating Patent Grant (CPG) between Japan and Cambodia, (2016).

[43] Memorandum of Understanding on the Cooperation of Intellectual Property between the Ministry of Commerce/National Committee for Intellectual Property Rights of the Kingdom of Cambodia (MOC/NCIPR) and the State Intellectual Property Office of The People's Republic Of China (SIPO) (2011).

[44] Memorandum of Understanding (MOU) on The Co-operation in Industrial Property between the Ministry Industry Handicraft (MIH) and The Intellectual Property Office of Singapore (IPOS), (January 20, 2015).

[45] Patent Cooperation Treaty (June 19, 1970).

[46] Memorandum of Understanding on The Patent Cooperation, (2019).

[47] Patent Worksharing arrangement (October 2020).

[48] Agreement on validation of European patents between the Government of the Kingdom of Cambodia and the European Patent Organisation., (1 March 2018).

[49] Prakas (declaration) on the Procedure for the Grant of Patents and Utility Model Certificates, (2007).

[50] The World Trade Organization. Intellectual Property: Least Developed Countries. Responding to least developed countries’ special needs in intellectual property 2021 [cited 2021 26th March 2021]. Available from: https://www.wto.org/english/tratop_e/trips_e/ldc_e.htm.

[51] Report of the Working Party on the Accession of Cambodia, (15 August 2003).

[52] Baker BK . Ending drug registration apartheid: Taming data exclusivity and patent/registration linkage. Am J Law Med. 2008;34(2-3):303–344. 18697696 10.1177/009885880803400209

[53] World Health Organization- Regional Office for South East Asia. Data exclusivity and other “trips-plus” measures 2017.

[54] UNAIDS. Cambodia 2022 [cited 2023 28th July 2023]. Available from: https://www.unaids.org/en/regionscountries/countries/cambodia.

[55] Vun MC Fujita M Rathavy T , et al. Achieving universal access and moving towards elimination of new HIV infections in Cambodia. J Int AIDS Soc. 2014;17(1):18905. 24950749 10.7448/IAS.17.1.18905 PMC4065309

[56] UNAIDS. Cambodia - Donor dependency in treatment interventions [cited 2023 28th July 2023]. Available from: https://hivfinancial.unaids.org/hivfinancialdashboards.html#.

[57] World Health Organization. Hepatitis C 24th June 2022 [cited 2022 11/8/2022]. Available from: https://www.who.int/news-room/fact-sheets/detail/hepatitis-c.

[58] Médecins Sans Frontières. Cambodia 2022 [cited 2022 11th August 2022]. Available from: https://msf.org.au/country-region/cambodia.

[59] Médecins Sans Frontières. Cambodia- Affordable drugs and new hepatitis C treatment method save lives 2017 [updated 21 August 2017; cited 2022 11/8/2022]. Available from: https://www.msf.org/innovating-hepatitis-c-treatment-cambodia.

[60] Zhang M O’Keefe D Iwamoto M , et al. High sustained viral response rate in patients with hepatitis C using generic sofosbuvir and daclatasvir in Phnom Penh, Cambodia. J Viral Hepat. 2020;27(9):886–895. 32358826 10.1111/jvh.13311

[61] Médecins Sans Frontières Access Campaign. Untangling the Web: HIV Medicine Pricing & Access Issues, 2020. Geneva, Switzerland, 2020.

[62] World Health Organization. Accelerating access to hepatitis C diagnostics and treatment: overcoming barriers in low-and middle-income countries: global progress report 2020. Accelerating access to hepatitis C diagnostics and treatment: overcoming barriers in low-and middle-income countries: global progress report 20202021.

[63] Senior M . Sovaldi makes blockbuster history, ignites drug pricing unrest. Nat Biotechnol. 2014;32(6):501. 24911474 10.1038/nbt0614-501

[64] Pollack A . Sales of Sovaldi, new Gilead hepatitis C drug, soar to $10.3 billion. New York Times, 2015, p. 3.

[65] Clinton Health Access Initiative. HCV Market Intelligence Report 2021 and Preliminary HBV Market Insights. 2021.

[66] Mermelstein S Stevens H . TRIPS To where? A narrative review of the empirical literature on intellectual property licensing models to promote global diffusion of essential medicines. Pharmaceutics. 2021;14(1):48. 35056944 10.3390/pharmaceutics14010048 PMC8779122

[67] MSF Access Campaign. Voluntary licenses and access to medicines. October 2020.

[68] Simmons B Cooke GS Miraldo M . Effect of voluntary licences for hepatitis C medicines on access to treatment: A difference-in-differences analysis. Lancet Global Health. 2019;7(9):e1189–e1e96. 10.1016/S2214-109X(19)30266-9 31362914

[69] Medicines Patent Pool. About Us [cited 2022 12/9/2022]. Available from: https://medicinespatentpool.org/who-we-are/about-us.

[70] Medicines Patent Pool. Progress & Achievements -Licences- Patents related to Darunavir. 2022 [cited 2022 12/9/2022]. Available from: https://medicinespatentpool.org/licence-post/patents-related-to-darunavir.

[71] Medicines Patent Pool. Progress & Achievements - Licences Lopinavir, Ritonavir (LPV/r) Paediatrics. 2022 [cited 2022 12/9/2022]. Available from: https://medicinespatentpool.org/licence-post/lopinavir-ritonavir-lpv-r-paediatrics.

[72] Medicines Patent Pool. Progress & Achievements - Licences Lopinavir, Ritonavir (LPV/r). 2022 [cited 2022 12/9/2022]. Available from: https://medicinespatentpool.org/licence-post/lopinavir-ritonavir-lpv-r.

[73] Ellen ‘t Hoen. COVID-19 and the comeback of compulsory licensing. 2020 [cited 2022 12/9/2022]. Available from: https://medicineslawandpolicy.org/2020/03/covid-19-and-the-come-back-of-compulsory-licensing/.

[74] Medicines Patent Pool. Progress & Achievements - Licences. Daclatasvir (DAC) 2022 [cited 2022 29/8/2022]. Available from: https://medicinespatentpool.org/licence-post/daclatasvir-dac.

[75] Gilead. Amended and Restated License Agreement. 2017.

[76] Medicines Patent Pool, ViiV Healthcare sign licence for the most recent HIV medicine to have received regulatory approval. [press release]. April 1 2014 April 1, 2014.

[77] Kunthear M . Hun Sen okays local production of COVID medication. The Phnom Penh Post. 12 March 2022.

[78] Ministry of Health Kingdom of Cambodia. List of Drug Products for Registration at the Ministry of Health, Cambodia- The Approval Licences. 2022, p. 3.

[79] Amin T Kesselheim AS . Secondary patenting of branded pharmaceuticals: A case study of how patents on two HIV drugs could be extended for decades. Health Aff (Millwood). 2012;31(10):2286–2294. 23048110 10.1377/hlthaff.2012.0107

[80] Kapczynski A Park C Sampat B . Polymorphs and prodrugs and salts (oh my!): An empirical analysis of “secondary” pharmaceutical patents. PLOS ONE. 2012;7(12):e49470. 10.1371/journal.pone.0049470 PMC3515607 23227141

[81] Moir HVJ . Exploring evergreening: Insights from two medicines. Aust Econ Rev. 2016;49(4):413–431.

[82] Yin N . Pharmaceuticals, incremental innovation and market exclusivity. Tulane University: Department of Economics and the Murphy Institute; 2015.

[83] Mendez SJ . Parallel trade of pharmaceuticals: The danish market for statins. Federal Reserve Bank of St Louis; 2016. 10.1002/hec.3551 28868645

[84] Duso T Herr A Suppliet M . The welfare impact of parallel imports: A structural approach applied to the German market for oral anti-diabetics. Düsseldorf: DICE Discussion Paper, No. 137, Düsseldorf Institute for Competition Economics (DICE); 2014. 10.1002/hec.3068 25139795

[85] Granlund D Koksal-Ayhan MY . Parallel imports and a mandatory substitution reform: A kick or a muff for price competition in pharmaceuticals? Eur J Health Econ. 2015;16(9):969–983. 25404013 10.1007/s10198-014-0646-9

[86] Rathod SK . Patent oppositions in India. In: Access to medicines and vaccines. Springer; 2022:151–182.

[87] The Patent Act (IPA), (1970).

