# Supplementary material for: Cambodia's Imminent Graduation from Least Developed Country Status: What Will be the Impact of the TRIPS Agreement on Access to HIV and Hepatitis C Medicines in Cambodia?
Source: Int J Soc Determinants Health Health Serv. 2024 Apr 2;54(3):295–308. doi: 10.1177/27551938241242602 (PMC11157998; doi:10.1177/27551938241242602)
Supplement: sj-docx-1-joh-10.1177_27551938241242602 - Supplemental material for Cambodia's Imminent Graduation from Least Developed Country Status: What Will be the Impact of the TRIPS Agreement on Access to HIV and Hepatitis C Medicines in Cambodia? [file sj-docx-1-joh-10.1177_27551938241242602.docx]

# Supplementary file 1

# ARV Scenario calculations

|  | 2020 | Scenario 1 | | Scenario 2 | | Scenario 3 | |
| --- | --- | --- | --- | --- | --- | --- | --- |
|  | number of adults on regimen | per unit | total with dosing | per unit | total with dosing | per unit | total with dosing |
| **1st Line** |  |  |  |  |  |  |  |
| Tenofovir/Lamivudine/Efaviranz - 300/300/400mg - TDF/3TC/EFV | 31923 | 0.19567 | 2279887.46 | 0.195667 | 2279887.46 | 0.182 | 2120644.89 |
| Tenofovir/Lamivudine/Dolutegravir - 300/300/50mg - TDF/3TC/DTG | 20939 | 0.17667 | 1350216.52 | 0.176667 | 1350216.52 | 0.17 | 1299264.95 |
| Other regimen (average cost of first line medicines) | 4888 | 0.18617 | 332143.67 | 0.186167 | 332143.67 | 0.176 | 314005.12 |
| **2nd Line** |  |  |  |  |  |  |  |
| Tenofovir/Lamivudine (300/300mg)+Atazavir/ritonavir (300/100mg) - (TDF/3TC+ATV/r) | 2748 |  |  |  |  |  |  |
| Tenofovir/Lamivudine (300/300mg)+(TDF/3TC) |  | 0.10667 | 106988.80 | 0.106667 | 106988.80 | 0.113 | 113341.26 |
| atazavir/ritonavir (300/100mg) |  | 0.433 | 434307.66 | 0.433 | 434307.66 | 0.45 | 451359.00 |
| Tenofovir/Lamivudine (300/300mg)+Lopinavir/ritonavir (200/50mg) - (TDF/3TC+LPV/r) | 120 |  |  |  |  |  |  |
| Tenofovir/Lamivudine (300/300mg) |  | 0.10667 | 4672.00 | 0.106667 | 4672.00 | 0.113 | 4949.40 |
| lopinavir/ritonavir (200/50mg) |  | 0.20833 | 36500.00 | 0.507 | 88826.40 | 0.163 | 28557.60 |
| Zidovudine/Lamivudine (300/150mg)+Atazavir/ritonavir (300/100mg) - AZT/3TC+ATV/r | 454 |  |  |  |  |  |  |
| Zidovudine/Lamivudine (300/150mg) |  | 0.088 | 29164.96 | 0.088 | 29164.96 | 0.088 | 29164.96 |
| atazavir/ritonavir (300/100mg) |  | 0.433 | 71752.43 | 0.433 | 71752.43 | 0.45 | 74569.50 |
| Zidovudine/Lamivudine (300/150mg)+Lopinavir/ritonavir (200/50mg) - AZT/3TC+LPV/r | 64 |  |  |  |  |  |  |
| Zidovudine/Lamivudine (300/150mg) |  | 0.088 | 4111.36 | 0.088 | 4111.36 | 0.088 | 4111.36 |
| lopinavir/ritonavir (200/50mg) |  | 0.20833 | 19466.67 | 0.507 | 47374.08 | 0.163 | 15230.72 |
| Abacavir/Lamivudine (600/300mg)+Atazanavir/ritonavir (300/100mg) - ABC/3TC+ATV/r | 527 |  |  |  |  |  |  |
| Abacavir/Lamivudine (600/300mg) |  | 0.295 | 56744.73 | 0.295 | 56744.73 | 0.307 | 59052.99 |
| atazavir/ritonavir (300/100mg) |  | 0.433 | 83289.72 | 0.433 | 83289.72 | 0.45 | 86559.75 |
| Abacavir/Lamivudine (600/300mg)+Lopinavir/ritonavir (200/50mg) - ABC/3TC+LPV/r | 78 |  |  |  |  |  |  |
| Abacavir/Lamivudine (600/300mg) |  | 0.295 | 8398.65 | 0.295 | 8398.65 | 0.307 | 8740.29 |
| lopinavir/ritonavir (200/50mg) |  | 0.20833 | 23725.00 | 0.507 | 57737.16 | 0.163 | 18562.44 |
| - Other regimen (average cost of 2nd line medicines) | 44 | 0.82572 | 13261.10 | 1.423056 | 22854.27 | 0.749667 | 12039.65 |
| **3rd Line** |  |  |  |  |  |  |  |
| Darunavir 600mg+TDF/3TC/DTG+Ritonavir 100mg | 32 |  |  |  |  |  |  |
| Darunavir 600mg |  | 0.883 | 20626.88 | 0.674 | 15744.64 | 0.674 | 15744.64 |
| TDF/3TC/DTG |  | 0.17667 | 2063.47 | 0.176667 | 1245.91 | 0.17 | 1985.60 |
| ritonavir 100mg |  | 0.23 | 2686.40 | 0.23 | 5804.96 | 0.114 | 1331.52 |
| Darunavir 600mg+Dolutegravir 50mg+Lamivudine 150mg+Ritonavir 100mg | 14 |  |  |  |  |  |  |
| Darunavir 600mg |  | 0.883 | 9024.26 | 0.674 | 2686.40 | 6888.28 |  |
| dolutegravir 50mg |  | 0.113 | 577.43 | 0.497 | 2539.67 | 0.087 | 444.57 |
| lamivudine 150mg |  | 0.07333 | 749.47 | 0.073333 | 749.47 | 0.037 | 378.14 |
| ritonavir 100mg |  | 0.23 | 1175.30 | 0.23 | 1175.30 | 0.114 | 582.54 |
| Darunavir 600mg+Dolutegravir 50mg+Ritonavir 100mg | 9 |  |  |  |  |  |  |
| darunavir 600mg |  | 0.883 | 5801.31 | 0.674 | 4428.18 | 0.674 | 4428.18 |
| dolutegravir 50mg |  | 0.113 | 371.21 | 0.497 | 1632.65 | 0.087 | 285.80 |
| ritonavir 100mg |  | 0.23 | 755.55 | 0.23 | 755.55 | 0.114 | 374.49 |
| Other regimen (average cost of 3rd line medicines) | 22 | 2.17911 | 17498.26 | 2.179111 | 17340.35 | 1.601333 | 12858.71 |
| Total |  |  | 4915960.24 |  | 5039461.20 |  | 4685456.33 |
| Percent of PLHIV covered by cost of scenario |  |  | 84% |  | 82% |  | 88% |

PLHIV = people living with HIV; ARV = antiretroviral

# DAA Scenario Calculations

| **Scenario 1 (baseline) - Current situation given LDC status and LDC TRIPS waiver** | | | |
| --- | --- | --- | --- |
|  | **DAA regime** | |  |
|  | Sofosbuvir | Daclatasvir | total |
| Cost of DAA regime per person (US$) | 52.5 | 36 |  |
| Number of people treated | 2,776 | 2,776 |  |
| Total cost of DAA regime by number of people treated | 145,740.00 | 99,936.00 | 245,676.00 |
| **Scenario 2- Introduction of patents when Cambodia graduates from LDC status and the LDC TRIPS waiver no longer applies** | | | |
| Cost of DAA regime per person (US$) | 690 | 546 |  |
| Number of people treated | 2,776 | 2,776 |  |
| Total cost of DAA regime by number of people treated | 1,915,440.00 | 1,515,696.00 | 3,431,136.00 |

DAA = direct acting antivirals; LDC = least developed country; TRIPS = Trade-Related Aspects of Intellectual Property Rights
